# Atypical aortic coarctation as a cause of a cardiomyopathy

**DOI:** 10.1007/s12471-015-0716-3

**Published:** 2015-06-02

**Authors:** F. Alsemgeest, O. Kamp, C.B. Marcu

**Affiliations:** Department of Cardiology, VU University Medical Center Amsterdam, De Boelelaan 1117, 1081 HZ Amsterdam, The Netherlands

**Keywords:** Cardiomyopathy, Atypical aortic coarctation, MR, CT, Echocardiography, Hypertension, Aorta, Coarctation, Aortic coarctation, Pulmonary hypertension

## Abstract

Atypical locations for aortic coarctation have been previously described. However, to our knowledge, no case has been described of a rapidly progressive dilated cardiomyopathy caused by an atypical coarctation, with a rapid normalisation of ventricular function after treatment.

A 40-year-old woman with a history of hypertension was referred to our hospital with progressive symptoms of exertional shortness of breath. On physical examination, her blood pressure was 140/90 mmHg in the right arm and 120/85 mmHg in the left arm. Auscultation of the heart revealed a grade III/VI loud systolic murmur, best heard in the suprasternal region and radiating to the back. Electrocardiogram and laboratory results were normal. Echocardiography showed a hypertrophic left ventricle with a poor left ventricular systolic function, restrictive diastolic function, moderate mitral regurgitation, pulmonary hypertension (estimated systolic pulmonary artery pressure (sPAP): 65 mmHg) and a dilated right ventricle. Continuous Doppler imaging of the proximal descending aorta showed a peak velocity of 4.75 m/s (corresponding to a peak pressure gradient of 90 mmHg; Fig. 1a). Cardiac magnetic resonance imaging was performed and confirmed the diagnosis of aortic coarctation, but in a rather atypical location involving the aortic arch (Fig. 1b). Computed tomography angiography furthermore revealed occluded left subclavian and left carotid arteries. Cardiac catheterisation showed normal coronary arteries and a mean pulmonary arterial pressure of 47 mmHg. The patient underwent stenting of the coarctation, which was unfortunately complicated by dissection of the descending thoracic aorta for which a conservative regimen was followed. One month after the procedure, the patient’s symptoms rapidly disappeared and her left ventricular function normalised.

**Fig. 1:**
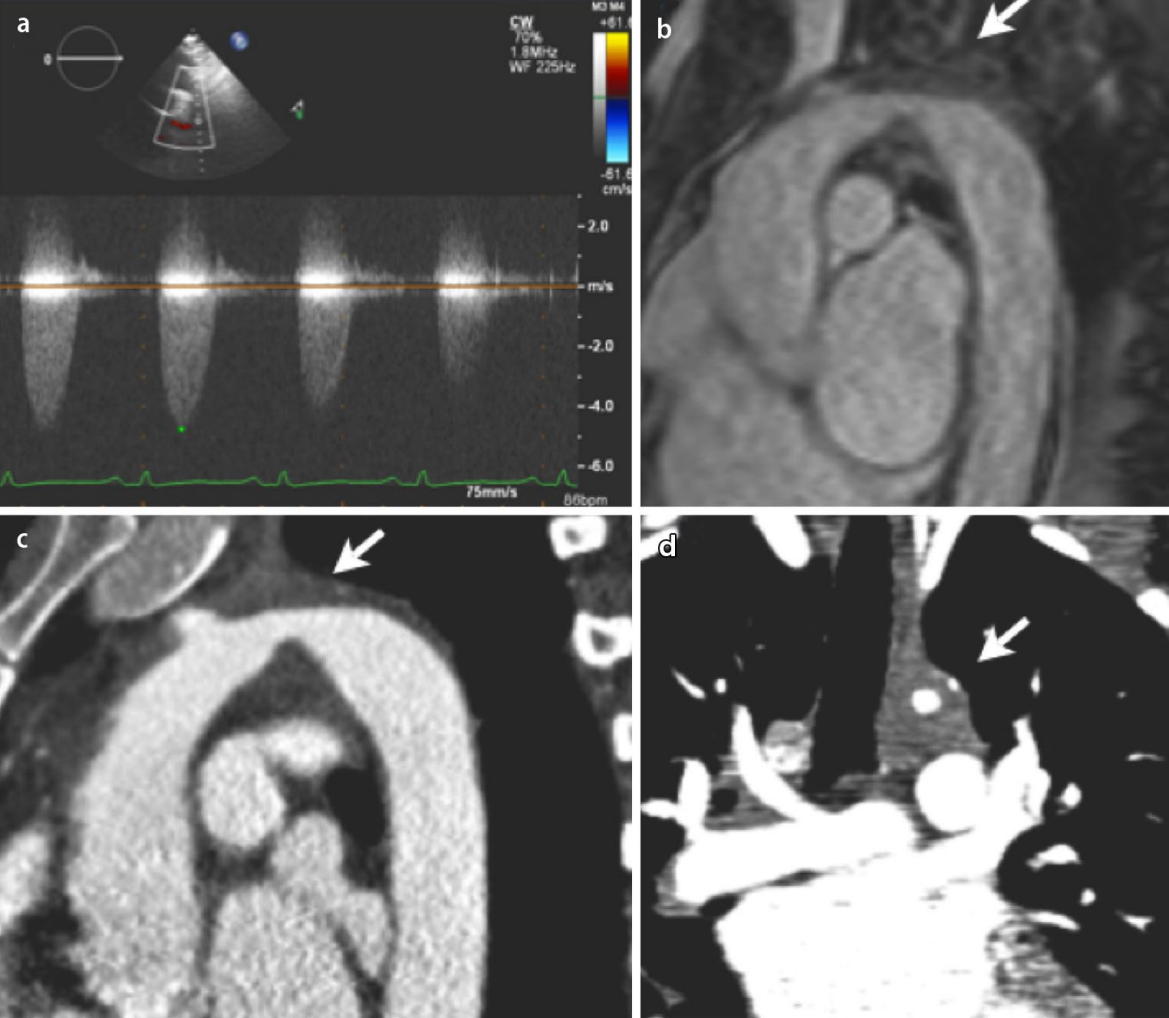
Imaging of the atypical aortic coarctation. **a** Doppler imaging of the descending aorta showing an elevated peak pressure gradient (90 mmHg). **b** Magnetic resonance and **c**, **d** computed tomographic images showing a coarction aorta on an atypical location in the aortic arch (*arrows*)

